# Corrigendum to “Plant-Derived Antioxidants in Disease Prevention”

**DOI:** 10.1155/2017/5092754

**Published:** 2017-05-31

**Authors:** Renata Szymanska, Pavel Pospisil, Jerzy Kruk

**Affiliations:** ^1^Department of Medical Physics and Biophysics, Faculty of Physics and Applied Computer Science, AGH University of Science and Technology, Reymonta 19, 30-059 Krakow, Poland; ^2^Department of Biophysics, Faculty of Science, Centre of the Region Haná for Biotechnological and Agricultural Research, Šlechtitelů 27, Palacký University, 78371 Olomouc, Czech Republic; ^3^Department of Plant Physiology and Biochemistry, Faculty of Biochemistry, Biophysics and Biotechnology, Jagiellonian University, Gronostajowa 7, 30-387 Krakow, Poland

In the article titled “Plant-Derived Antioxidants in Disease Prevention” [1], affiliation number three was incomplete. The corrected affiliation is shown above.
